# Enhancement of Optical Telecommunication Bands: Pr^3+^-Doped Halide Phosphate Glasses Display Broadband NIR Photoluminescence Emission

**DOI:** 10.3390/ma15196518

**Published:** 2022-09-20

**Authors:** Bilel Charfi, Kamel Damak, Ramzi Maâlej, Mohammed S. Alqahtani, Khalid I. Hussein, Ali M. Alshehri, Abdulrahman M. Hussain, Bozena Burtan-Gwizdala, Manuela Reben, El Sayed Yousef

**Affiliations:** 1 LaMaCoP, Faculty of Sciences of Sfax, University of Sfax, Sfax 3018, Tunisia; 2 Department of Radiological Sciences, College of Applied Medical Sciences, King Khalid University, Abha 61421, Saudi Arabia; 3 BioImaging Unit, Space Research Centre, Department of Physics and Astronomy, University of Leicester, Leicester LE1 7RH, UK; 4 Department of Medical Physics and Instrumentation, National Cancer Institute, University of Gezira, Wad Medani 2667, Sudan; 5 Physics Department, Faculty of Science, King Khalid University, Abha 61413, Saudi Arabia; 6 Institute of Physics, Cracow University of Technology, ul. Podchorazych 1, 30-084 Cracow, Poland; 7 Faculty of Materials Science and Ceramics, AGH–University of Science and Technology, al. Mickiewicza 30, 30-059 Cracow, Poland; 8 Research Center for Advanced Materials Science (RCAMS), King Khalid University, Abha 61413, Saudi Arabia

**Keywords:** phosphate glasses, rare earth, spectroscopic, Judd–Ofelt, photoluminescence

## Abstract

In the optical energy gap, visible and near-IR emission of halide phosphate glasses with a composition of 40P_2_O_5_-30ZnO-20LiCl-10BaF_2_ in mol% doped with 3.5 × 10^4^ ppm Pr_2_O_3_, referred to as PZLBPr, were synthesized. The UV-VIS-NIR and spectroscopic properties of these glasses were also predicted. The current glasses had broadband emission photoluminescence covering a wavelength range of 1250 to 1700 nm when excited at 455 nm. These bands for near-infrared emission luminescence relate to the transitions ^1^G_4_ → ^3^H_5_, ^1^D_2_ → ^1^G_4_, and ^3^H_4_ → ^3^F_3_, ^3^F_4_ in the optical telecommunication window. The significant PL emission wideband was caused by the radiative transition from Pr^3+^: ^1^D_2_ to ^1^G_4_. At 445 nm excitation, these glasses exhibited emission bands that corresponded to blue/reddish orange spectral ranges in visible ranges. The prepared glass has a high lasing quality factor (Ω_4_/Ω_6_ = 0.9), high optical energy (4.72 eV), and quantum efficiency = 87.3% with FWHM = 156 nm of transition emission from the ^1^D_2_ → ^1^G_4_ level. As a result, broadband near infrared optical amplifiers can be fabricated from the prepared glasses.

## 1. Introduction

The C-, S-, E-, and O bands may be enhanced with the use of rare earth RE ions including Er^3+^, Tm^3+^, and Ho^3+^ [1,2,3,4,5]. It has been investigated whether the co-doped RE ions may improve population inversion and increase bandwidth [2,3] to avoid a number of undesirable parameters, such as frequent cross-relaxation and concentration quenching occurring. In order to overcome the restrictions of the RE ion multi-doped system, an efficient RE ion single-doped ultra-broadband signal amplification medium must be developed. The commercial Er^3+^-doped fiber amplifiers (EDFA) based on silicate glass have low linewidths, which reduce NIR transmission [5,6,7,8].

In consideration of this, new glass host matrices containing rare earths with extended lifetimes for signal amplification in optical telecommunications must be searched for. Pr^3+^, a trivalent rare earth ion, displays broad near-infrared luminescence as a result, which is required for optical fiber amplifiers that operate in the O-, E-, S-, C-, and L-bands [1,2] in the 1.2 to 1.7 m wavelength range of 1.2 to 1.7 μm.

Studies in the past have shown the optical amplification potential of Pr^3+^ ions existing in a variety of inorganic glasses, particularly those with low-loss NIR transmission windows [3,8]. Low-loss NIR luminescence in the ^1^D_2_ → ^1^G_4_ transition of Pr^3+^ transition correlates to super broadband NIR luminescence.

In heavy metal oxide glasses, the luminescence spectroscopy of Pr^3+^ ions was studied in [9,10,11,12,13]. As a result of energy transfer from Pr^3+^ to Yb^3+^ ions, we recently described the intense NIR emission at 1000 nm with a down-conversion mechanism under excitation of 445 nm [14]. The down-conversion processes in Pr^3+^/Yb^3+^ co-doped devices that convert a blue photon into two NIR photons have also been reported. Trivalent Yb^3+^ emission at 1000 nm may be captured by silicon solar cells without any losses (1.05 eV) because its single ^2^F_5/2_ excited state is close to the silicon band gap [15].

Fiber lasers and optical amplifiers have revealed the phosphate glasses to be an excellent product because of their superior thermal stability, flexibility, and mechanical strength [6,7,16]. For the first time, near-IR luminescence has been seen in 40P_2_O_5_-30ZnO-20LiCl-10BaF_2_ glasses with single doped 3.5 × 10^4^ ppm of Pr^3+^, which we describe in this paper. As an additional factor, the Pr^3+^ ligand field was modified by the addition of certain halides (F^−^ and Cl^−^). Moreover, we estimated the spectroscopic parameters viz. branching ratio, radiative lifetime, spontaneous emission probability, and quantum efficiency of the ^1^D_2_ level of Pr^3+^. The results show that the produced glass may be used to create broadband fiber amplifiers that operate in optical communications.

## 2. Experimental Work

Glass composed of 40P_2_O_5_-30ZnO-20LiCl-10BaF_2_ in mol% doped with 3.5 × 10^4^ ppm Pr_2_O_3_ is referred to as (PZLBPr), which has a high homogeneity. Following the melt quenching method, we used a muffle furnace to melt the chemical at 1150 °C for one hour after stirring it in a platinum crucible. A copper mold was then used to cast the molten chemical combination. The annealing procedure started with a 2 h heat treatment of the quenched glass at 440 °C to remove any strain from the produced glasses. To investigate the amorphous characteristics of produced glasses, the powder X-ray diffraction pattern (XRD-Philips PW (1140) diffractometer and copper target (K = 1.54) were used.

A gas pycnometer was used to determine the density of the glass (Model: UltraPyc 1200 e). The density of the PZLBPr glass sample was measured to be 3.837 ± 0.00015 g/cm^3^. The optical absorption spectra of the glasses were determined in the wavelength range of 190–2500 nm using a UV-VIS-NIR spectrophotometer (JASCO, V-570). The short pulse stimulation was carried out using an optical parametric oscillator operated by a third harmonic of a Nd:YAG laser, and the luminescence decay curves were obtained as a result. Using an Optron Dong Woo fluorometer system, the photoluminescence spectra were measured [17].

The following equation may be used to determine the Pr^3+^ ions concentration [13]:

(1) $$N_{{\mathrm{Pr}}^{3+}}=2\frac{3.5\rho A_{\upsilon }}{100M}$$

where *M* is the PZLBPr glass’s molecular weight and *A_ν_* is the Avogadro’s number. In this PZLBPr glass, the dopant Pr^3+^ concentration is *N*_Pr_^3+^ = 1.3628 × 10^27^ ions/m^3^.

## 3. Results and Discussion

**Figure 1:**
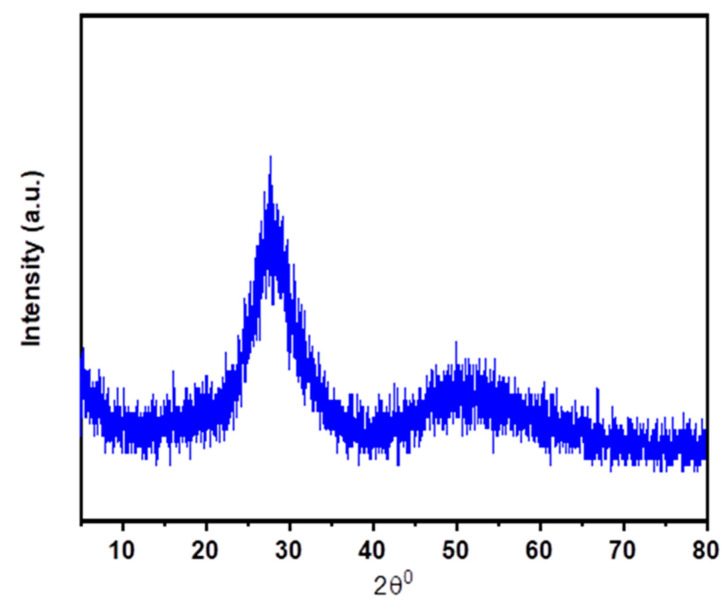
XRD profile of prepared glasses.

**Figure 2:**
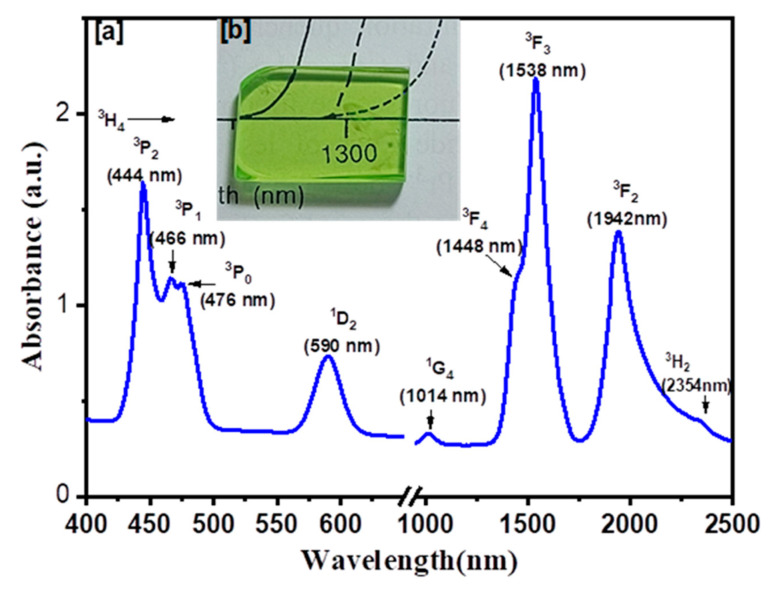
(**a**) PZLBPr glass absorption spectra, with each peak’s transition state specified; (**b**) photograph of the prepared glasses after annealing.

To determine whether the produced glasses are amorphous or crystalline, XRD analysis was performed. Figure 1 displays the XRD patterns of the samples of prepared glasses. The X-ray amorphous patterns can be observed in the data, and because the indicated crystalline phase had not developed the amorphous nature was anticipated. According to the photo of prepared glasses PZLBPr in Figure 2b, they were transparent and homogenous. According to Figure 2a, the PZLBPr sample’s UV-Vis-NIR absorption spectrum exhibits peaks at wavelengths around 444, 466, 476, 590, 1014, 1538, 1942, and 2354 nm that correspond to transitions from the ground state of ^3^H_4_ to excited states of ^3^P_2_, ^3^P_1_, ^3^P_0_, ^1^D_2_, ^1^G_4_, ^3^F_4_, ^3^F_3_, ^3^F_2_, and ^3^H_6_, respectively. The transition from ^3^H_4_ to ^3^F_3_ has the highest peak, whereas the transfer from ^3^H_4_ to ^1^G_4_ has the lowest peak. The occurrence of all these peaks is in agreement with findings regarding other glasses doped with Pr^3+^ [18,19,20,21,22].

**Figure 3:**
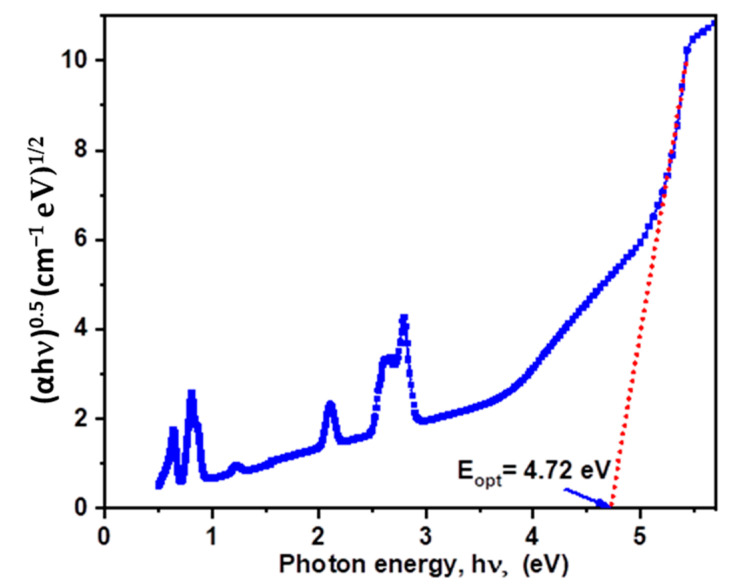
Relation between (*h*αν)^0.5^ vs. *hα* of PZLBPr glasses.

The value of optical energy gap, *E_opt_*, is calculated using the basic relation established for amorphous materials by Mott and Davis [23] based on the absorption coefficient (*α*) and the photon energy (*hv*) as follows:

(2) $$\left(\alpha h\nu \right)=C{\left(h\nu -E_{opt}\right)}^{m}$$

where *m* changes based on the interband transition process and C is a constant. As shown in Figure 3, the best fit to the observed data showed that *m* = 2 indicated the indirect allowed transition in the gap of the prepared glass, which means that it is a suitable method for the prediction of the optical energy gap and optical properties of glass materials.

The indirect transitions allowed in inorganic glasses are represented by a straight line at (*αhν*)^1/2^ = 0, with an hν axis in Equation (2), which illustrates the prepared glasses (see Figure 3). The results obtained showed that the value of *E_opt_* is 4.72 ± 0.01 eV for PZBLPr glass. It has the highest value of *E_opt_* compared to tellurite glass and other glass doped with the Nd^3+^/Yb^3+^ system that have been reported previously [24,25,26,27]. This is due to the presence of Pr^3+^ ions, which leads to an increase in both the width of the localized state in the forbidden gap and the density of electrons, therefore, increasing the probability of electronic transition from the valence band to the conduction band.

The incorporation (F, Cl) improved the phosphate glass matrix by increasing the solubility of rare earth, which means that the glass network can form more than one electric dipole environment for rare-earth ions. Consequently, the fluorescence line shape and radiative and non-radiative rates may be created in hybrid glass structures. Furthermore, the F and Cl ions in the prepared glass reduce the OH^−^ and Pr^3+^ ions, which enter the lattice sites by replacing OH ions. Furthermore, the halide ions F^−^ and Cl^−^ can lead to an increase in the ionicity and consequently decrease the covalency of the network structure of matrix glasses, which results in a larger optical energy gap when compared to oxide phosphate glasses reported in the literature [28,29,30]. This can be considered an advantage because the wide band gap of this glass can form an excellent medium with doping or co-doped rare earth to produce superior materials that can be used in optical telecommunication at ultrabroad NIR.

**Figure 4:**
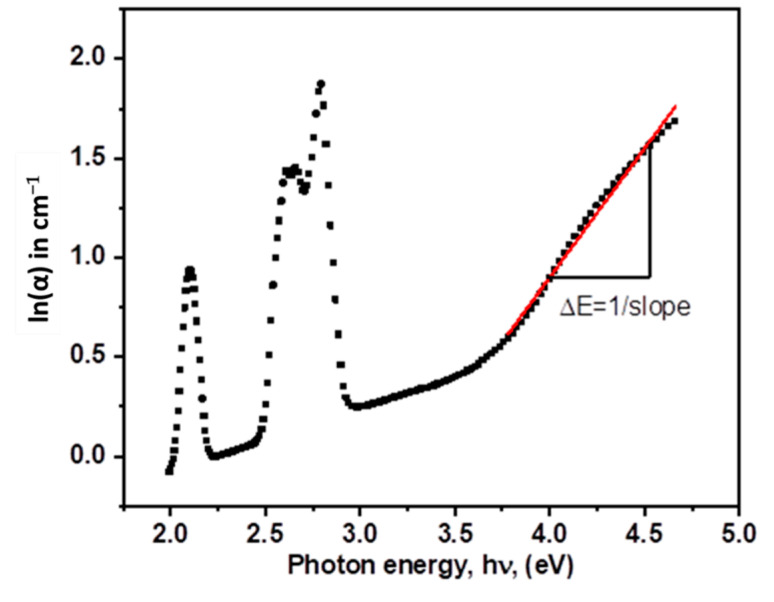
The relation between ln(α) *vis. hν* of PZLBPr glasses.

Here, we determine the Urbach energy, *E_Ub_*, using the relationship between the photon energy (*hν*) and optical absorption coefficients in glasses established by [23].

(3) $$\alpha \left(\nu \right)=A\exp\left(\frac{h\nu }{E_{Ub}}\right)$$

where *A* is a constant and *E_Ub_* is the Urbach energy corresponding to the extent of the tail of localized states in the band gap. The phonon-assisted indirect electronic transitions are responsible for *E_Ub_*. The reciprocals of the slopes of the linear component of the *ln*(*α*) vis. *hν* curves in the lower photon energy areas were used to calculate the *E_Ub_* value (0.07 eV) (see Figure 4).

The Judd–Ofelt (JO) analysis was used to calculate the spectroscopic characteristics of this glass system using the absorption intensities of Pr^3+^-doped PZLBPr. A basic description of the JO analysis is provided here. The literature [31,32,33] includes all the applications of the JO model.

The formula below yields the measured line strength $S_{\exp}(J\to J^{\prime })$ of a particular band:

(4) $$S_{\exp}(J\to J^{\prime })=\frac{9n}{{\left(n^{2}+2\right)}^{2}}\cdot 4\pi \varepsilon_{0}\cdot \frac{3c.h.(2J+1)}{8\pi^{3}e^{2}}\cdot \frac{2.303}{N.l.\lambda }\cdot \Gamma -n.S_{md}(J\to J^{\prime })$$

where *l* is the studied sample’s thickness (6.22 mm), *c* is the speed of light, *h* is Planck’s constant, *e* is the elementary charge, *J* is the angular momentum of the initial state, *N* is the density of ions, *λ* is the mean wavelength of the absorption bands, and Γ=∫OD(λ). dλ is the experimental integrated optical density in the wavelength range. This density can be determined by calculating the total area under the absorbed band.

For many transitions of the Pr^3+^ ion, the contribution of the magnetic dipole, $S_{md}$, to the observed line strength is generally small [19,22,34,35]. Because of this, the magnetic-dipole contribution was neglected in the prepared glasses. Table 1 depicts the results of the line-strength calculations and intensity measurements for the selected transitions.

On the other hand, in the Judd–Ofelt theory, the line strength $S_{cal}(J\to J')$ between the initial state *J* characterized by (*S*, *L*, *J*) and the final state *J*′ given by (*S*′, *L*′, *J*′) can be calculated using the following expression [31,32,33].

(5) $$S_{cal}(J\to J^{\prime })=\sum_{t=2,4,6}\Omega_{t}{\left|\langle SLJ\left\|U^{\left(t\right)}\right\|S^{\prime }L^{\prime }J^{\prime }\rangle \right|}^{2}$$

where Ω*_t_* (*t* = 2, 4, 6) denotes the doubly reduced matrix elements and *t* represents the Judd–Ofelt parameters. *||U^(t)^||*^2^ (*t* = 2, 4, 6) represent the reduced matrix elements utilized for the absorption transitions of this Pr^3+^-doped glass presented in Table 1 and includes the *||U*^(*t*)^*||*^2^ matrix elements estimated by Carnall et al. [36] and evaluated by Kaminiskii [37].

The values of the three JO parameters were obtained by fitting the measured and computed line strengths of the absorption transitions using Equations (4) and (5). To calculate the Ω*_t_* (*t* = 2, 4, 6) parameters for the PZLBPr sample under study, a least square fitting of values $S_{\exp}$ to $S_{cal}$ was used.

When using all absorption transitions, it was found that the resulting Ω_2_ parameter was negative, which from the definition is not acceptable. This controversial result is noticed in many compounds with Pr^3+^ ions [38,39,40].

Several methods have been proposed to resolve this problem, namely, the use of the modified JO theory [38,41]. The modified JO theory developed by Kornienko et al. [22,41,42,43] considers higher-order contributions in the forced electric dipole matrix elements; the resulting calculated line strength is provided using:

(6) $$S_{cal}=\sum_{t=2,4,6}\Omega_{t}\left[1+2\alpha (E_{J}+E_{J^{\prime }}-2E_{4f}\right]\times {\left|\langle SLJ\left\|U^{\left(t\right)}\right\|S^{\prime }L^{\prime }J^{\prime }\rangle \right|}^{2}$$

*E_J_*_′_, *E_J_*, and *E*_4*f*_ are the energies of the upper, lower states, and barycenter of the 4*f* configuration, respectively, the parameter *α* is used as a fitting parameter.

The Kornienko et al. [42] modified JO theory was used in this study with the value of the parameter *α* fixed to 10^−5^ cm as it is often estimated for Pr^3+^glasses [22,43]. The procedure of the modified JO theory did not solve the problem and the Ω_2_ value is still negative.

Another approach to overcoming this problem consists of excluding one of the absorption transitions from the fitting procedure [40,44,45,46,47]. For this Pr^3+^ sample, the exclusion of the ^3^H_4_ → ^3^F_4_ transition from the computation is investigated. Notably, absorptions due to transitions from the ground state up to ^3^F_4_ and ^3^F_3_ levels strongly overlap. In several studies, those two levels are combined into one: the (^3^F_4_, ^3^F_3_ → ^3^H_4_) transition. Furthermore, a close observation of the absorption transition matrix elements of the Pr^3+^ ion shows that the Ω_2_ parameter depends essentially on the ^3^H_4_ → ^3^F_2_ transition because of the high value of $\langle {}^{3}H_{4}{\left\|U^{\left(2\right)}\right\|}^{2}{{}_{}}^{3}F_{2}\rangle$.

Without accounting for the ^3^H_4_ → ^3^F_4_ transition, the Ω_t_ numeric calculation produces an acceptable result. Table 2 presents JO parameters of Pr^3+^ in PZLBPr glass and Pr^3+^-doped in other hosts. The calculated Judd–Ofelt parameters are in good agreement with literature values for Pr^3+^-doped glasses [19,20,38,48]: similar trends (Ω_2_ < Ω_4_ < Ω_6_) have been found in PPbKANPr [19], TeO_2_-PbF_2_-AlF_3_-PrF_3_ (TPA) [38], ASL [44], PTBPr [43], and Ca_5_(PO_4_)_3_F [48] glasses.

The acquired Ω*_t_* values are utilized to obtain the rms deviation and recalculate the transition line strengths *S_cal_* of the absorption bands using Equation (3). In our work, the rms deviation was determined to be 1.4868 × 10^−25^ m^2^. The significant mixing between the 4*f* and 5d orbitals may account for the increased rms difference between the estimated and observed oscillator strengths in the case of Pr^3+^ ions. The covalency, ionic composition, and symmetry of the RE ion’s site are all factors connected to the Ω_2_ parameter. In contrast, values Ω_4_ and Ω_6_ are closely connected to the basicity, hardness, and dielectric properties of the glass samples [19]. With an increase in the hardness of the host matrices’ surroundings, Ω_6_ decreases. In the present PZLBPr glass, the trend from Ω_2_ < Ω_4_ < Ω_6_ showed that there was more asymmetry and less covalency within the PrO group. The stimulated emission cross-section for the laser active medium is predicted by the spectroscopic quality factor χ = Ω_4_/Ω_6_, which is found to be around 0.9 for this PZLBPr glass and similar to the value for the PPbKANPr0.5 sample [19].

According to Table 2, the range of the spectroscopic quality factors for Pr^3+^ doped in various hosts is between 0.20 and 1.64.

For a change from a higher *J*′ to a lower *J*-multiplet, the spontaneous emission probability *A_JJ′_* is computed as follows:

(7) $$A_{J\to J^{\prime }}=\frac{1}{4\pi \varepsilon_{0}}\frac{64\pi^{4}e^{2}\upsilon^{3}n{\left(n^{2}+2\right)}^{2}}{27h(2J+1)}S_{cal}$$

$S_{cal}$ is the corresponding emission line strength calculated from Equation (6) using the value of Ω*_t_*. In addition, the following formulas might be used to determine the radiative lifetime for electric dipole transitions between an excited state (*J*) and the lower-lying terminal manifolds (*J*′):

(8) $$\tau =\frac{1}{\sum_{J^{\prime }}A_{J\to J^{\prime }}}$$

Over all final states (*J*′), the total of *τ* is calculated and the sum is taken over all final states *J*′.

The fluorescence branching ratio, *β*, is an important statistic for the laser designer since it describes the potential of achieving a stimulated emission from a certain transition and predicts the relative strength of lines from specified excited states.

(9) $$\beta =A_{J\to J^{\prime }}\cdot \tau$$

Table 3 shows the PZLBPr glass system’s spectroscopic properties; ($A_{J\to J^{\prime }}$), (*τ*), and (*β*) of excited states ^3^H_4_, ^3^H_5_,^3^H_6_, ^3^F_2_, ^3^F_3_, ^3^F_4_, ^1^G_4_, ^1^D_2_, ^3^P_0_, ^3^P_1_, and ^3^P_2_ of Pr^3+^ ions in the PZLBPr glasses.

**Figure 5:**
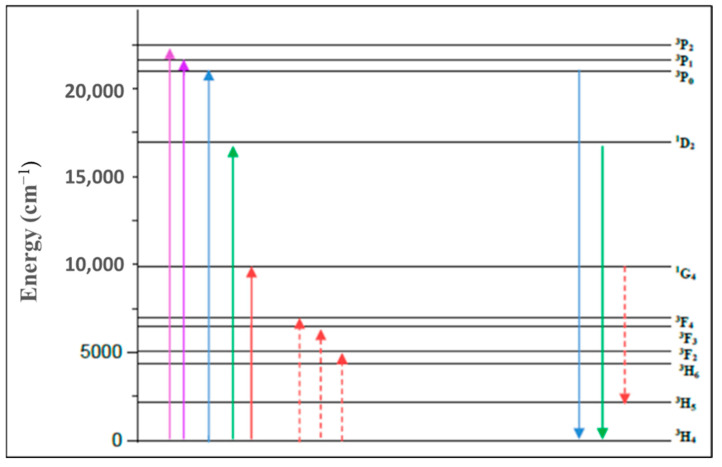
Energy level diagram of Pr^3+^ ions.

It is observed that the lifetime trend decreases as ^3^H_6_ >> ^3^F_2_ > ^3^F_4_ > ^1^G_4_ > ^3^F_3_ > ^1^D_2_ > ^3^P_1_ > ^3^P_0_~^3^P_2_. There is a decreasing tendency of branching ratios for the emission transitions, ^3^F_2_ → ^3^H_4_ > ^3^F_3_ → ^3^H_4_ > ^3^F_4_ → ^3^H_4_ > ^3^P_0_ → ^3^H_4_ > ^1^G_4_ → ^3^H_5_ > ^1^D_2_ → ^3^H_4_ > ^3^P_1_ → ^3^H_4_ > ^3^P_2_ →^3^H_4_. The branching ratios of ^3^P_0_ → ^3^H_4_ (blue) and ^1^D_2_ → ^3^H_4_ (red) transitions are estimated to be 67% and 47%, respectively. Thus, the blue and red/orange emissions are expected to be dominant among all visible transitions. An interesting result is that the branching ratio for the ^1^G_4_ → ^3^H_5_ transition is larger than the ^1^G_4_ → ^3^H_4_ transition. Precisely, ^1^G_4_→ ^3^H_5_ presents an elevated fluorescence branching ratio (β ~ 67%) characterizing the lasing potential of this transition, in the near-infrared region. Since the ^1^G_4_ level has a sufficiently large energy gap with respect to the next lower level ^3^F_4_, it emits via purely radiative relaxations an infrared fluorescence emission as shown in Figure 5. It should be noted that the ^1^G_4_ level is efficiently pumpable at 1.01μm, especially that high population inversion could be obtained, given that the excited initial state, (^3^H_5_), relaxes directly to the ground state seeing the higher branching ratio of the ^3^H_5_ → ^3^H_4_ transition.

**Figure 6:**
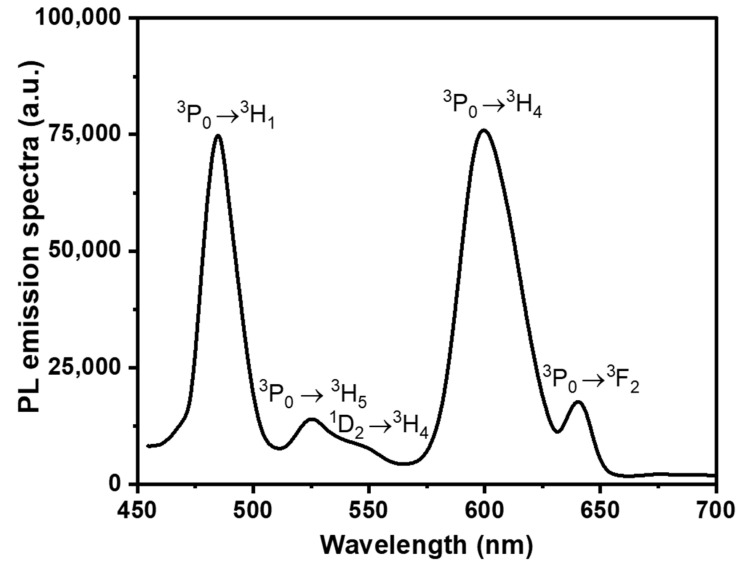
VIS PL emission spectra of PZLBPr under excitation 445 nm.

The PL emission spectra of the prepared glass excited at 445 nm are shown in Figure 6. The ^3^P_0_ → ^3^H_4_, ^3^P_0_ → ^3^H_5_, ^1^D_2_ → ^3^H_4_, ^3^P_0_ → ^3^H_6_, and ^3^P_0_ → ^3^F_2_ transitions of Pr^3+^ are responsible for the emission bands shown at 485, 525, 550, 600, and 640 nm, respectively. The type of glass host has a significant influence on the intensity of emission bands in the blue and reddish orange range. Two prominent transition peaks in the visible emission spectrum are attributed to ^3^P_0_ → ^3^H_4_ (blue) and ^1^D_2_ → ^3^H_4_/ ^3^P_0_ → ^3^H_6_ (red/orange). The higher sub-levels of ^3^P_0_ energy may be absorbed by a surrounding Pr^3+^ in the ground state when they are close together, which causes a decrease in the higher energy levels of ^3^P_0_. The spectrum of ^3^P_0_ ↔ ^3^H_4_ overlaps the high-energy emission and low-energy absorption sides.

Furthermore, compared to the ^3^P_0_ →^3^H_4_ transition, the emission intensity increase from the ^1^D_2_ state is significantly lower. Because of the population or depopulation of the ^1^D_2_ state of Pr^3+^ ions in host glasses is significantly influenced by multi-phonon relaxation and cross relaxation. Pr^3+^ ions undergo the ^3^P_0_ → ^3^H_4_ transition as the excitation energy is changed from the lower ^3^P_0_ state to the ^1^D_2_ state, resulting in reddish orange luminescence. Under 445 nm excitation, Bo Zhou et al. [5] examined the visible PL spectra of Pr^3+^ doped fluorotellurite glasses. They determined that the mission bands at wavelengths of 490, 528, 611, and 643 nm, respectively, correspond to the Pr^3+^ transitions of (^3^P_1_, ^3^P_0_) → ^3^H_4_, ^3^P_0_ → ^3^H_5_, (^3^P_1_, ^3^P_0_) →^3^H^6^, and ^3^P_0_ → ^3^F_2_, respectively. Our data are excellent agreement with those of other glasses described in Refs. [5,54,55,56]. As a consequence, we can conclude that PZLBPr is a high phonon glass that can occupy the energy gap between the visible range ^3^P_0_ and ^1^D_2_ states of Pr^3+^ ions and the red/orange emission caused by ^1^D_2_ → ^3^H_4_.

**Figure 7:**
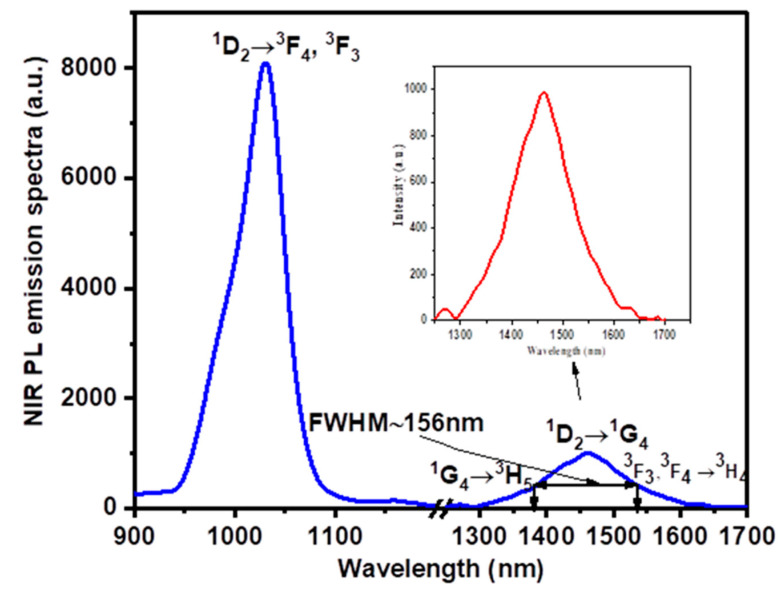
NIR PL emission spectra of PZLBPr under excitation at 445 nm.

Figure 7 displays the NIR luminescence emission spectra of PZLBPr-produced glass excited by 445 nm (^3^P_2_) at wavelengths between 850 and 1700 nm. Observing that the (^1^D_2_ → ^3^F_3_, ^3^F_4_) and (^1^G_4_ → ^3^H_5_) and (^1^G_4_ → ^3^H_5_) transitions of Pr^3+^ are responsible for the near-infrared luminescence bands at 1030 and 1350 nm is significant for optical fiber amplifiers operating in the second telecommunication window [53,55,56]. The ^1^D_2_ → ^1^G_4_ transition of Pr^3+^ is responsible for the strong NIR luminescence band with a center wavelength of around 1460 nm. The ultra-broadband near-infrared fluorescence band ^3^F_3_, ^3^F_4_ → ^3^H_4_ transition at around 1685 nm also contributed. As can be seen in Figure 7, the full-width at half-maximum (FWHM) for the equivalent ^1^D_2_**γ** ^1^G_4_ transition emission is 156nm, which indicates that the PZLBPr glass is very suitable for broadband amplification of E-, S-, C-, and L-bands.

According to X. Liu et al. [1], the associated ^1^D_2_ → ^1^G_4_ transition emission has a 140 nm full-width at half-maximum (FWHM), which enables Pr^3+^-doped NZPGT glasses attractive for broadband amplification covering the entire E-, S-, C-, and L-bands. The ultra-broadband emission at 1.48 μm has a peak in intensity at 1476 nm. Additionally, previous findings in [1,2,57,58] suggest that Pr^3+^-doped borate, phosphate, and silicate glass fiber amplifiers operating in the fifth optical communications window may be useful (1380–1525 nm): the PZLBPr glass is a better choice for usage in optical communication devices as it has a greater FWHM of the transition emission of ^1^D_2_ → ^1^G_4_ (156 nm) than the present glass does.

**Figure 8:**
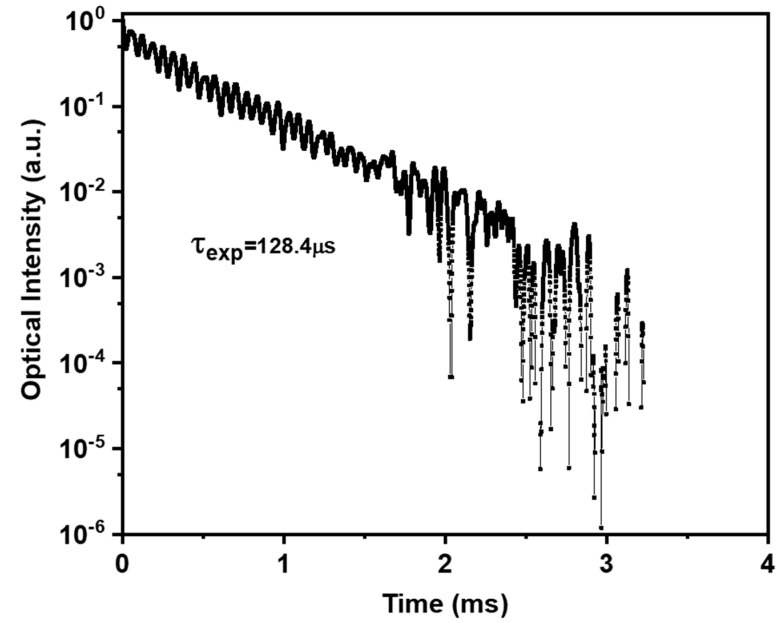
The Pr^3+^: ^1^D_2_ fluorescence decay level of PZLBPr under excitation at 445 nm.

The lifetime values are calculated using the formula: $\tau_{exp}=\int t\varepsilon \left(t\right)/\varepsilon \left(t\right)$, where the decay curve is dependent on time, as shown in [14,17]. Figure 8 depicts the PL decay of stimulated levels. The radiative lifetime, estimated to be 147.1 μs, and the experimental lifetime, estimated to be 128.4 μs by fitting the fluorescence decay curve, are used to calculate the quantum efficiency, $\eta_{eff}=\frac{\tau_{exp}}{\tau_{rad}}$, for a luminescence level of ^1^D_2_ to ^1^G_4_. The near-infrared emission from the ^1^D_2_ level is efficient for creating optical signal amplification in a specific optical communications band, as shown by the quantum efficiency of the Pr^3+^: ^1^D_2_ level, which is equivalent to 87.3%. Together, these findings demonstrate that PZBLPr glass’s specific optical communications band may effectively produce optical signal amplification via the near-infrared emission at the ^1^D_2_ level.

## 4. Conclusions

The incorporation of halide ions (F^−^ and Cl^−^) with Pr^3+^ ions into the present phosphate matrix leads to improvement of the high value of the optical energy gap compared to some phosphate glasses reported previously. The valuation of Judd–Ofelt parameters of PZBLPr glasses achieved the following: (Ω_2_ = 0.0181 × 10^−20^ cm^2^, Ω_4_ = 1.641 × 10^−20^ cm^2^, Ω_6_ = 1.816 × 10^−20^ cm^2^). The trend of Ω_2_ < Ω_4_ < Ω_6_ that occurred in the studied glasses indicated higher asymmetry and lower covalency between Pr-O groups. In addition, the halide ions led to a decrease in the covalency otherwise an increase in the ionicity of the structure of PZBLPr glasses. The large quantum efficiency of Pr^3+^:^1^D_2_ (=87.3%) with full-width at half maximum of 156 nm was reported in PZLBPr glass. Therefore, based on this analysis, it can be concluded that PZLBPr glass has potential for broadband near-IR functioning and can have its communications transmission window increased at 445 nm.

## Figures and Tables

**Table 1:** The results of the line-strength calculations and intensity measurements for the transitions performed.

	λ (nm)	ν (cm^−1^)	||U^2^||^2^	||U^4^||^2^	||U^6^||^2^	Γ (nm·cm^−1^)	S_exp_ (10^−24^ m^2^)	S_cal_ (10^−24^ m^2^)
^3^H_4_ → ^3^P_2_	445	22471	0	0.0362	0.1355	19.11	0.54705	0.30554
^3^P_1_	462	21652	0	0.1707	0	7.51	0.20720	0.28026
^3^P_0_	476	21012	0	0.1728	0	14.61	0.39129	0.28371
^1^D_2_	590	16956	0.0026	0.017	0.052	10.31	0.22271	0.12241
^3^F_3_	1541	6490	0.0654	0.3469	0.6983	215.68	1.78360	1.83910
^3^F_2_	1958	5106	0.5089	0.4032	0.1177	137.06	0.89182	0.88521

**Table 2:** Comparison of the PZLBPr glass Judd–Ofelt parameters (Ω_t_ × 10^−20^ cm^2^) with those of other systems.

System	Ω_2_	Ω_4_	Ω_6_	Trend	χ
PZLBPr [Present Work]:	0.018	1.641	1.816	Ω_2_ < Ω_4_ < Ω_6_	0.90
PPbKANPr0.5 [19]	1.51	18.03	19.81	Ω_2_ < Ω_4_ < Ω_6_	0.91
Phosphate [49]	4.19	4.29	6.40	Ω_2_ < Ω_4_ < Ω_6_	0.67
ZNBBP [50]	1.7	3.06	4.72	Ω_2_ < Ω_4_ < Ω_6_	0.64
BPGBPr [51]	0.70	2.96	7.03	Ω_2_ < Ω_4_ < Ω_6_	0.42
Oxyfluoride [52]	0.66	12.49	3.17	Ω_2_ < Ω_6_ < Ω_4_	3.94
Ca_5_(PO_4_)_3_F [48]	0.32	1.59	3.82	Ω_2_ < Ω_4_ < Ω_6_	0.41
LaF_3_ [53]	0.12	1.77	4.78	Ω_2_ < Ω_4_ < Ω_6_	0.37
LiPrP_4_O_12_ [53]	1.82	2.83	6.54	Ω_2_ < Ω_4_ < Ω_6_	0.43
YAlO_3_ [53]	2.00	6.00	7.00	Ω_2_ < Ω_4_ < Ω_6_	0.85
LiYF_4_ [53]	0.00	8.07	7.32	Ω_2_ < Ω_6_ < Ω_4_	1.10
Oxy–Fluoride [21]	0.13	4.09	6.33	Ω_2_ < Ω_4_ < Ω_6_	0.64
TPA(n = cst) [38]	0.48	1.39	13.5	Ω_2_ < Ω_4_ < Ω_6_	0.10
TPA (n ≠ cst) [38]	0.92	1.85	6.61	Ω_2_ < Ω_4_ < Ω_6_	0.27
TeO_2_-Li_2_CO_3_-Pr_2_O_3_ [38]	3.81	5.81	4.1	Ω_2_ < Ω_6_ < Ω_4_	1.41
PTBPr [43]	3.07	3.36	8.68	Ω_2_ < Ω_6_ < Ω_4_	0.38

**Table 3:** The spectroscopic parameters of the PZLBPr glass system.

Transition		Wavelength (nm)	A (s^−1^)	τ (ms)	β (%)
^3^P_2_ →	^3^H_4_	431.77	4393.6	0.040	17.6
	^3^H_5_	476.0	5891.9		23.7
	^3^H_6_	532.72	7053.9		28.3
	^3^F_2_	550.54	3420.7		13.7
	^3^F_3_	597.18	2776.2		11.1
	^3^F_4_	613.28	1078.3		4.3
	^1^G_4_	755.32	268.29		1.1
	^1^D_2_	1716.4	26.99		0.1
	^3^P_0_	5647.2	0.023		0.0
	^3^P_1_	8671.9	0.013		0.0
^3^P_1_ →	^3^H_4_	454.39	5762.6	0.076	43.9
	^3^H_5_	503.64	0		0.0
	^3^H_6_	567.59	1391.1		10.6
	^3^F_2_	587.86	47.37		0.4
	^3^F_3_	641.35	2435.6		18.6
	^3^F_4_	659.95	3141.5		24.0
	^1^G_4_	827.39	338.42		2.6
	^1^D_2_	2139.9	0.2739		0.0
	^3^P_0_	16190	0		0.0
^3^P_0_ →	^3^H_4_	467.51	16068	0.041	67.4
	^3^H_5_	519.81	150.98		0.6
	^3^H_6_	588.21	3329.6		14.0
	^3^F_2_	610.01	139.41		0.6
	^3^F_3_	667.80	0.00		0.0
	^3^F_4_	687.99	3539.2		14.8
	^1^G_4_	871.95	609.13		2.6
	^1^D_2_	2465.8	0.10		0.0
^1^D_2_ →	^3^H_4_	576.89	737.95	0.147	47.8
	^3^H_5_	658.66	14.84		1.0
	^3^H_6_	772.48	294.00		19.1
	^3^F_2_	810.52	291.04		18.9
	^3^F_3_	915.82	42.40		2.7
	^3^F_4_	954.23	49.17		3.2
	^1^G_4_	1349.0	113.84		7.4
^1^G_4_ →	^3^H_4_	1007.9	18.33	3.096	5.7
	^3^H_5_	1287.1	218.72		67.7
	^3^H_6_	1807.6	71.32		22.1
	^3^F_2_	2030.6	2.19		0.7
	^3^F_3_	2852.3	2.42		0.7
	^3^F_4_	3261.1	9.96		3.1
^3^F_4_ →	^3^H_4_	1458.8	199.48	4.995	99.7
	^3^H_5_	2126.5	0.00		0.0
	^3^H_6_	4055.7	0.00		0.0
	^3^F_2_	5381.7	0.69		0.3
	^3^F_3_	22,753.0	0.01		0.0
^3^F_3_ →	^3^H_4_	1558.8	401.43	2.489	99.9
	^3^H_5_	2345.7	0.00		0.0
	^3^H_6_	4935.5	0.00		0.0
	^3^F_2_	7049.1	0.20		0.01
^3^F_2_ →	^3^H_4_	2001.4	127.81	7.823	100
	^3^H_5_	3515.5	0.00		0.0
	^3^H_6_	16,460.0	0.00		0.0
^3^H_6_ →	^3^H_4_	2278.4	11.59	86.286	100
	^3^H_5_	4470.3	0.00		0.0
^3^H_5_ →	^3^H_4_	4646.6	0.00		0.0

## Data Availability

Not applicable.
